# A Model of Demasking and Hydrolysis of Peptide Bonds During Tryptic Digestion of β-Casein and β-Lactoglobulin

**DOI:** 10.3390/molecules31020225

**Published:** 2026-01-09

**Authors:** Mikhail M. Vorob’ev

**Affiliations:** A.N. Nesmeyanov Institute of Organoelement Compounds, Russian Academy of Sciences, 28 ul. Vavilova, 119991 Moscow, Russia; mmvor@ineos.ac.ru

**Keywords:** digestion mechanisms, trypsin, β-lactoglobulin, β-casein, demasking, peptide release, in silico proteolysis

## Abstract

The prediction of polypeptide chain fragmentation during digestion (proteolysis) of protein substrates by trypsin was carried out for globular β-lactoglobulin (β-LG) and micellar β-casein (β-CN). Despite significant differences in the protein structures of these substrates, the concentrations of peptide fragments are calculated as functions of time or degree of hydrolysis using the same equations derived from the general proteolysis model. This model considers the opening of protein substrates in the course of proteolysis, the so-called demasking process, and the subsequent hydrolysis of specific peptide bonds at different rates determined by the amino acid sequence of hydrolyzed sites. The use of this model for in silico prediction of proteolysis is discussed. An algorithm for calculating demasking rate constants based on the experimental distribution of peptide fragments is presented. The calculated concentration dependence on the degree of hydrolysis of peptide bonds was compared with the experimental data for the intermediate and final peptide fragments of β-LG and β-CN. The predicted and experimental concentration curves for the final products were compared based on their curvatures. For both substrates, the predicted redistribution of peptide fragments in the course of proteolysis was found to be consistent with the experimental one.

## 1. Introduction

Proteolysis is widespread in living systems and is also used in the food industry, biotechnology, pharmaceuticals, cosmetology, etc. Proteolysis is quite difficult to study, as it is a continuous process of interconversion of various peptide fragments, i.e., the composition of the reaction mixture changes significantly during the hydrolysis of peptide bonds. The concentrations of the final products of proteolysis and of sufficiently long intermediate peptides containing not-yet-hydrolyzed enzyme-specific bonds change with the time of hydrolysis, and it is difficult to predict the concentration dependence for all these peptide fragments [1,2,3].

A practically important task is to obtain biopeptides from proteins using proteolysis. To obtain the final products of proteolysis, proteolysis must be completed. Conversely, intermediate biopeptides can be obtained by limited proteolysis over a specified time interval, after which the process should be stopped to avoid further hydrolysis of peptide bonds. To determine this time interval or the corresponding degree of peptide bond hydrolysis, proteolysis modeling must be used to assist in the experimental approach, which itself requires a large number of experiments [4,5,6,7,8,9,10,11,12].

Using the rate constants of peptide bond hydrolysis and other kinetic parameters, it is possible to calculate the time dependence of the concentrations of final and intermediate products using chemical kinetics methods [4,9,10]. To simplify this task, several approaches have been proposed that allow for a reduction in both the number of rate constants and the number of peptide fragments taken into account when modeling the proteolysis process [1,9].

Unlike low-molecular-weight substrates of proteases, many peptide bonds in proteins are initially inaccessible to the enzyme, as they are located within the protein globule or, for example, in the casein micelle. Disruption of the protein globule or micelle in the first step of proteolysis initiates the process of opening the internal peptide bonds for successful enzymatic attack [1,4,13]. This process, called demasking, eliminates steric hindrances to the enzyme so that the active site of the enzyme and the amino acid residues of the peptide substrate can fully interact. After demasking, hydrolysis of various peptide bonds occurs with different hydrolysis rate constants. During this step of proteolysis, peptide bonds are hydrolyzed according to their specificity, determined by the amino acid sequence of the substrate polypeptide chain.

We have previously described a method for determining demasking rate constants using fluorescence spectroscopy, which allows the rate of substrate opening during proteolysis to be estimated from the red shift of fluorescence accompanying proteolysis [13]. The use of this spectral method, in addition to the traditional determination of the rate of hydrolysis of peptide bonds, allowed us to analyze the interdependence of the demasking and hydrolysis processes within the framework of the two-step proteolysis model [4,13]. The demasking rate constants obtained by the fluorescence method were used to model the release of peptides during proteolysis of β-lactoglobulin (β-LG) by trypsin [9].

Modeling of peptide release during proteolysis of β-LG by trypsin was recently performed using a proteolysis model that considers both one- and two-stage demasking of peptide bonds without their secondary masking [9]. In addition to assumptions regarding the proteolysis mechanism, some simplifications were made in the calculation procedure itself. It was assumed that there is no secondary masking [14], the size of the intermediate peptide fragments is limited by two enzyme-specific peptide bonds within them, which can be subjected to further hydrolysis. The formation of some minor peptide fragments, which are formed very slowly, was neglected [9].

It is believed that the hydrolysis of proteins depends on their spatial structure, at least at the initial stage of the process [1,15,16]. Traditionally, to identify these dependencies, proteolysis of globular milk proteins, including β-LG and structurally disordered caseins, most often β-casein (β-CN) [1,14,17], is compared.

The major protein in bovine whey is the small globular protein β-LG (55–60%) [18,19]. The monomeric β-LG is made up of 162 amino acid residues (~18.3 kDa) and is stabilized by 2 disulfide bonds [19]. Its secondary structure was predicted mainly as β-sheet (50%) [20]. β-LG and its enzymatic hydrolysates have high functional and nutritional properties. The hydrolysates of β-LG contain a lot of biopeptides that can be used as biologically active additives in therapeutic nutrition and cosmetics [21].

Casein micelles are colloidal complexes of proteins and salts, and their main biological function is to transport sparingly soluble calcium phosphate in liquid form to infants [22]. They have four major protein species, termed αs1-, αs2-, β- and κ-caseins [22]. β-CN is the most hydrophobic one within the group of caseins, which constitutes about 45% of the casein of bovine milk [18]. It is a ~24 kDa single polypeptide chain, which consists of 209 amino acid residues [23]. Since β-CN does not contain disulfide bonds, it has no tertiary structure, but some regions of the polypeptide chain have secondary structure. β-CN has a hydrophobic C-terminus and a hydrophilic negative N-terminal region [24]. Caseins are known to be easily hydrolyzed by proteases due to their conformational flexibility and the abundance of the enzyme-accessible peptide bonds. β-CN is of interest because of its nutritional importance and utility as a drug delivery vehicle [25,26]. The hydrolysates of β-CN, as well as β-LG, are rich in biologically active peptides [27,28].

Since demasking of peptide bonds predetermines their hydrolysis, it can be assumed that the demasking rate constants can be calculated from the distribution of the resulting peptide fragments. Previously, the sum of the concentrations of the peptides obtained during the hydrolysis of the analyzed peptide bond was used to determine the selectivity parameter of this bond in the hydrolyzed protein [29,30,31]. In this paper, we expand on this approach and present a method for estimating the demasking rate constants by analyzing changes in the sums of the concentrations of some peptides over hydrolysis time. Grouping such peptides and calculating the sum of their concentrations to estimate the demasking rate constants corresponds to the proposed proteolysis model [9].

Recently, computer-aided search for bioactive peptides in enzymatic hydrolysates of proteins of various origins has become increasingly popular, which requires the development of reliable algorithms for in silico proteolysis [32,33,34]. The existing approaches are primarily aimed at predicting peptide fragments that can potentially be released during enzymatic hydrolysis of various protein substrates by various proteases. Prediction of concentration dependence for peptide fragments and the degree of hydrolysis required to obtain target peptides has not been conducted. These aspects of in silico proteolysis are of particular interest to us, as the proteolysis model we are developing is capable of solving such problems.

The aim of this work is to develop a simpler method for determining the demasking rate constants than the previously proposed fluorescence spectroscopy method [9,13]. Previously, demasking rate constants were estimated from the fluorescence shift during proteolysis, but this is now performed using experimental data on peptide release. Using a proteolysis model previously tested for β-LG proteolysis by trypsin [9], the proteolysis of β-CN is analyzed here, with demasking rate constants determined by the new method. In addition to a detailed demonstration of the method for calculating concentration curves, our goal is to compare the model predictions with the experiment for these two substrates, which have different protein structures.

## 2. Results

### 2.1. Proteolysis Model

**Figure 1:**
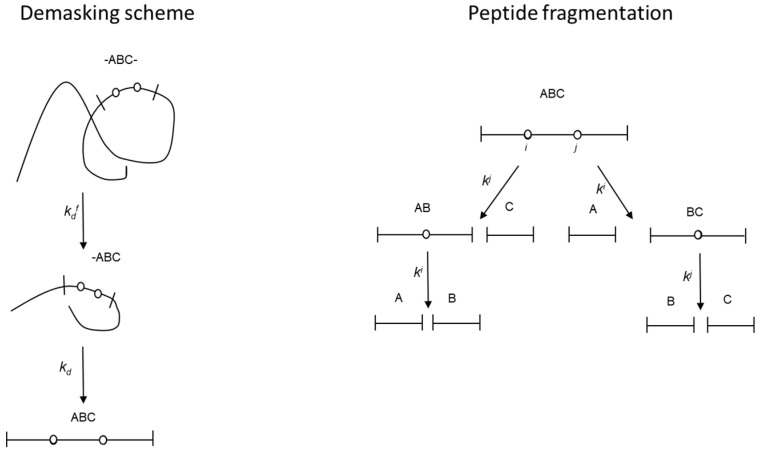
General scheme of proteolysis, including two stages of demasking with demasking rate constants $k_{d}^{f}$ and *k_d_* (**left side**) and subsequent hydrolysis of the demasked peptide bonds *k^j^* and *k^i^* (**right side**).

The same scheme of polypeptide chain cleavage was used here as in the previous work devoted to modeling the release of peptides during proteolysis [9]. The basic principles of the model are presented in Figure 1. The hydrolysis of masked peptide bonds is preceded by a two-stage demasking process, which involves conformational changes in the polypeptide chains and makes the peptide bonds accessible to the enzyme. The kinetic scheme in Figure 1 shows the peptide fragment ABC formed by the hydrolysis of the most rapidly hydrolyzed peptide bonds at the beginning of proteolysis. It consists of three relatively short peptides, A, B and C, which include only peptide bonds that are nonspecific for the enzyme. These peptides are linked by the enzyme-specific peptide bonds A-B and B-C, indexed *i* and *j*, respectively.

The left side of Figure 1 shows that these peptide bonds are not hydrolyzed until their demasking is complete. The right side of Figure 1 shows how these demasked bonds are hydrolyzed with hydrolysis rate constants *k^j^* and *k^i^* to form the corresponding peptide fragments.

Thus, Figure 1 shows the transformation of the peptide chain -ABC- into a partially demasked peptide fragment -ABC (ABC-) in the first stage of demasking, with the demasking rate constant $k_{d}^{f}$. Thus, Figure 1 shows the transformation of the -ABC- peptide chain into a partially demasked fragment -ABC (ABC-) in the first demasking step. Then, at the second stage of demasking, a completely demasked peptide fragment ABC is formed with demasking rate constant *k_d_*. After demasking, the fragmentation of the ABC trimer occurs as a result of hydrolysis of the peptide bonds *i* and *j* with the formation of peptide fragments AB, BC, A, B, and C.

### 2.2. Application of the Fragmentation Scheme to the Proteolysis of β-CN by Trypsin

To specify the general scheme (Figure 1) for the proteolysis of β-CN by trypsin, we collected data that were previously published for this enzyme–substrate pair (Table 1). These parameters include the selectivity and initial rate of hydrolysis of the enzyme-specific peptide bonds Lys-X and Arg-X in β-CN. Both of these characteristics correlate fairly well with each other, and they allow ranking various peptide bonds by their rate of hydrolysis. From these data, it follows that peptide bonds with indexes 99, 105, and 169 are the most rapidly hydrolyzed peptide bonds, while bonds 1, 25, 32, 48, and 202 are the most slowly hydrolyzed peptide bonds, the hydrolysis of which is neglected within the framework of the considered model. The difference in selectivity between the most slowly and most rapidly hydrolyzed peptide bonds was 1–2 orders of magnitude (Table 1). This difference is very large, and the assignment of peptide bonds to these two categories does not change with changes in proteolysis conditions, although the selectivity of different peptide bonds changes slightly with changes in proteolysis conditions for a given enzyme–substrate pair [7,30].

The remaining peptide bonds (28/29, 97, 107, 113, 176, and 183) are considered initially masked, and the model must quantify how these peptide bonds are demasked and hydrolyzed. Thus, during proteolysis of β-CN by trypsin, the ABC fragments are f(1–99), f(106–169), and f(170–209), and the fragment f(100–105) is a rapidly forming peptide that does not contain enzyme-specific peptide bonds.

The peptide bonds assigned to the group of the most slowly hydrolyzed bonds are considered non-hydrolyzed in our simplified approximation. When analyzing experimental data, the concentrations of peptide fragments resulting from the hydrolysis of such bonds are summed with the concentrations of the peptides from which they originated. For example, the concentrations of fragments f(184–202) and f(203–209) are added to the concentration of peptide f(184–209), since bond 202 is considered the most slowly hydrolyzed peptide bond (Table 1). The sum obtained in this way is considered the concentration of fragment f(184–209).

In the previous analysis of β-LG proteolysis by trypsin, demasking of two ABC fragments occurred by the two-stage demasking mechanism and of one trimer by the one-stage demasking mechanism [9]. Proteolysis of β-CN by trypsin is a multi-step process that includes, in addition to hydrolysis, the association of intermediate peptides and the formation of nanoparticles during proteolysis [35]. Due to the complexity of this process, we consider demasking all three trimers—f(1–99), f(106–169) and f(170–209)—using the two-stage demasking scheme. Due to the complexity of this process and based on the results of fitting using Equation (13), we considered demasking all three trimmers f(1–99), f(106–169) and f(170–209) using the two-stage demasking scheme.

### 2.3. Estimation of Rate Constants for Demasking and Hydrolysis

Like the selectivity parameter [29], $k_{d}^{f}$ was determined using a simple exponential equation for the most rapidly hydrolyzed peptide bond (Equation (11)).

In this study, a new approach to estimate the demasking rate constants was used, which differs from the fluorescence spectroscopy method [13]. This approach utilizes the dependence of the sum of the concentrations of specific peptide fragments on the hydrolysis time. To calculate the demasking rate constant *k_d_*, Equation (12), derived from the material balance equations, was used. This equation is given in Section 4 for the sum of the concentrations of [*ABC*], [*AB*], and [*A*], corresponding to the balance of A units. To track the balance of C units, it is necessary to calculate the sum [*ABC*] + [*BC*] + [*C*]. Fitting using Equation (12) yielded the *k_d_* value for each ABC trimer. The found *k_d_* values then allowed us to determine the rate constants of peptide bond hydrolysis using Equation (13), which we previously derived for the concentrations of peptide bonds in proteolysis with demasking [1,14].

This approach to estimating the rate constants required for the simulations according to the scheme in Figure 1 was applied to the proteolysis of β-CN by trypsin (Table 2). For comparison with the previous result on peptide release during proteolysis [9], this method was also applied to the proteolysis of β-LG by trypsin (Table 2).

### 2.4. Simulation of Peptide Release During Proteolysis

**Figure 2:**
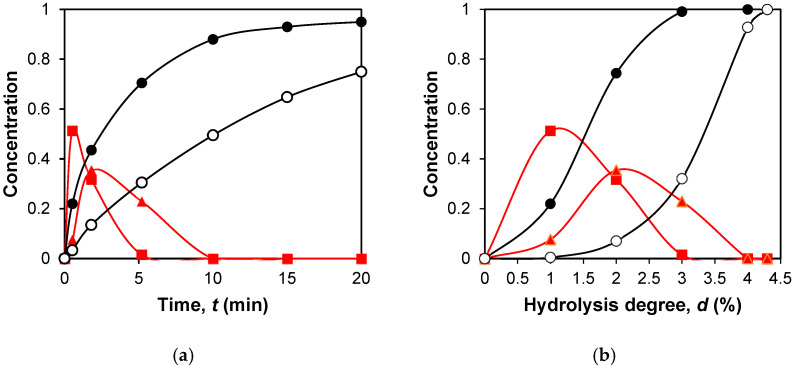
Simulation of peptide release during tryptic hydrolysis of β-CN: (**a**) calculated dependence of peptide concentrations on the hydrolysis time for the intermediate peptides f(1–99) (■) and f(30–99) (▲), and for the final products of proteolysis f(170–176) (●) and f(106–107) (○); (**b**) calculated dependence of peptide concentrations on the degree of hydrolysis of peptide bonds for the same peptide fragments.

Figure 2a shows an example of the calculated dependence of peptide concentrations on the hydrolysis time for some intermediate (f(1–99) and f(30–99)) and final (f(170–176) and f(106–107)) peptide fragments. The intermediate peptide products (ABC, AB, and BC) are formed first and then disappear due to the hydrolysis of internal enzyme-specific peptide bonds. Final products (A, B, and C) only accumulate, since they do not contain internal enzyme-specific peptide bonds. The time required for demasking of peptide bonds leads to the fact that the concentrations of proteolysis products may not increase immediately with the onset of proteolysis but after a lag phase [1].

The concentrations of peptide fragments can also be represented as functions of the degree of hydrolysis of peptide bonds achieved at a given time (Figure 2b), since the dependence of the degree of hydrolysis on time is known. Representing concentration curves as functions of the degree of hydrolysis is more convenient for determining the mechanisms of peptide release during proteolysis. The transition from time to the degree of hydrolysis does not change the shape of the curves for intermediate products but changes the dependencies for the final peptides. For these, the curves for rapidly released final peptides remain convex, while those for slowly released peptides become concave.

The parameter *d_r_*, the degree of hydrolysis of peptide bonds at which the major part of a given peptide is released, was calculated for each of the intermediate products using the following equation:

(1) $$d_{r}=\sum_{i=1}^{6}d_{i}\times C(d_{i})/\sum_{i=1}^{6}C(d_{i})$$

where *C*(*d_i_*) are the molar concentrations of peptide fragment determined at six hydrolysis degrees *d_i_* (0, 1.5, 3, 4.5, 6, 7.9% for β-LG and 0, 1, 2, 3, 4, 4.3% for β-CN). Thus, *d_r_* is defined as the weighted average of 6 degrees of hydrolysis, and the relative concentrations of a given intermediate peptide corresponding to these degrees of hydrolysis are the weighting factors.

For the final peptides, the parameter *n*, which characterizes the dependence of their concentrations on the degree of hydrolysis of peptide bonds *C*(*d_i_*), was calculated using the following equation:

(2) $$C(d_{i})=a{\left(d_{i}/d_{\max}\right)}^{n}$$

where *a* is a constant factor, *d_max_* is the maximal hydrolysis degree (7.9% for β-LG and 4.3% for β-CN), and *n* is the exponent of the power function. Thus, we compared the kinetic curves for the final products simply by comparing the values of parameter *n*, which reflects the curvature of the curves. The proteolysis parameters *d_r_* and *n* were introduced by Equations (1) and (2) in such a way that they did not depend on the absolute molar concentrations of the peptide components, but depended on the relative ones.

**Figure 3:**
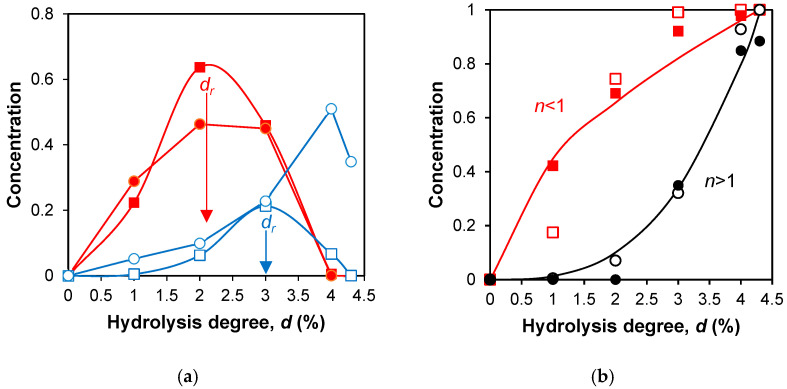
Comparison of the calculated release of β-CN peptides with the experimental one: (**a**) calculated (■) and experimental (●) curves for peptide fragment f(106–169), ABC. Calculated (□) and experimental (○) curves for peptide fragment f(106–113), AB; (**b**): calculated (■) and experimental (□) concentration dependence for peptide f(170–176), A. Calculated (○) and experimental (●) concentration dependence for peptide f(114–169), C. Solid lines correspond to Equation (2) with *n* = 0.55 for peptide f(170–176) (red) and *n* = 2.98 for peptide f(114–169) (black).

The examples of calculated and experimental concentration dependence for the intermediate (Figure 3a) and final (Figure 3b) fragments are presented. Figure 3a shows the release of the intermediate trimeric peptide ABC during proteolysis and the subsequent formation of the intermediate dimeric peptide AB from it. Theoretically, *d_r_* for the ABC fragment is expected to be smaller than for the AB or BC fragments, which is confirmed by the location of the peaks in Figure 3a.

Figure 3b shows the concentration curves for the final peptides. For a rapidly forming final peptide, the curve is convex and *n* is less than 1, while for a slowly forming final peptide, the curve is concave and *n* is greater than 1.

Table 3 presents the parameters of the concentration curves for all peptide fragments studied, namely *d_r_* for intermediate peptides and *n* for final peptides. The parameters *d_r_* and *n* were calculated according to Equations (1) and (2) using simulated and experimental data on peptide concentrations. The published peptide concentrations were obtained by tryptic proteolysis of β-LG and β-CN in the experiments carried out under identical conditions, including temperature and pH [7]. The concentrations of peptide fragments were calculated using Equations (3)–(8) for the same degrees of hydrolysis as in the experiment.

**Figure 4:**
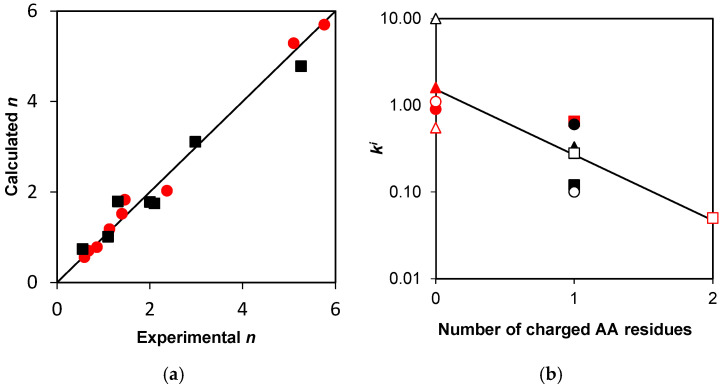
Estimation of the model parameters: (**a**) correlation between experimental and calculated *n* for the final products of β-LG (●) and β-CN (■). The straight line shows the expected proportionality coefficient of 1; (**b**) Dependence of the hydrolysis rate constants *k^i^* on the number of charged amino acid residues in positions P_2_ and P_−2_ for peptide bonds in β-LG 14 (●), 40 (▲), 83 (■), 91 (○), 124 (∆), 135 (□) and in β-CN 28/29 (●), 97 (▲), 107 (■), 113 (○), 176 (∆), 183 (□). A linear correlation between lg(*k^i^*) and number of polar groups with slope of −0.76 ± 0.18 (*r*^2^ = 0.632) is shown.

The comparison of the *n* values for the simulated and experimental concentration curves obtained for both β-LG and β-CN proteolysis is shown in Figure 4a. The coefficient of proportionality between the experimental and calculated *n* values was 0.999 ± 0.038 (*r*^2^ = 0.9998) for β-LG (Figure 4a) with the expected coefficient of 1. Thus, the agreement between the simulation results and the experiment was very good when using the parameter *n* for comparison. The proportionality coefficient between the experimental and calculated *d_r_* values was 0.68 ± 0.07 (*r*^2^ = 0.87), indicating an underestimation of the simulated parameter for the intermediate peptide fragments with high *d_r_*.

The proportionality coefficient between the experimental *n* and that calculated using the previous quantitative assessment of rate constants [9] was 0.77 ± 0.10 (*r*^2^ = 0.892), while in this study, it is 0.96 ± 0.04 (*r*^2^ = 0.989) for all points in Figure 4a. Thus, the use of the method described here for estimating the demasking and hydrolysis rate constants allows for a more accurate prediction of the release of proteolysis products using the same proteolysis model that we proposed earlier [9]. This confirms the correctness of the main ideas of the model based on the consideration of the processes of the peptide bond demasking before their hydrolysis and the difference in hydrolysis rate for the different peptide bonds.

Despite the differences in the structure of β-LG and β-CN substrates, calculations of the concentration dependence and the parameters of *d_r_* and *n* were performed using the same algorithm and yielded results consistent with experiment.

### 2.5. Prospects for In Silico Proteolysis

The possibility of predicting the hydrolysis rate constants *k^i^* for various protein substrates based on the analysis of their amino acid sequences is illustrated in Figure 4b. The total number of charged amino acid residues in positions P_2_ and P_−2_ was chosen as the key parameter. It has been previously shown that the presence of charged residues (E, D, R, K) in these positions significantly reduces the selectivity parameter of proteolysis by trypsin [7]. Figure 4b shows that lg(*k^i^*) decreases with increasing numbers of charged groups in P_2_ and P_−2_ positions for the studied peptide bonds in both substrates. Constructing more reliable correlation equations requires a larger volume of experimental data obtained for a larger number of protein substrates. It is also possible to consider amino acid residues in P_1_ and P_−1_ positions, as in predicting peptide bond hydrolysis in low-molecular-weight substrates.

In our approach, the equation linking amino acid sequence to reactivity must be sought specifically for the hydrolysis rate constants *k^i^*, since they reflect the hydrolysis step of proteolysis. According to the model, to find a correlation equation corresponding to the given proteolytic enzyme, all demasked regions of the polypeptide chain and their corresponding *k^i^* must be considered, regardless of their affiliation with specific protein substrates. Therefore, we plotted the hydrolysis constants for β-LG and β-CN on a single graph (Figure 4b).

The demasking rate constant $k_{d}^{f}$ of 0.46 for β-LG, determined from the opening of the protein globule, is expectedly lower than that for β-CN ($k_{d}^{f}$ = 3.5), which is a disordered coil of the polypeptide chain with more accessible peptide bonds. Therefore, the ratio of the demasking rate constants of β-CN and β-LG determined here is 7.6 under the proteolysis conditions published in [7]. It is appropriate to compare proteolysis processes carried out at the same concentrations of substrate and enzyme when the temperature, pH and other environmental conditions remain unchanged. Previously, the ratio of the demasking rate constants for proteolysis of β-CN and β-LG by trypsin was determined from the fluorescence shift [13], and this ratio was equal to 12.9 (kII(β-CN)/kII(β-LG) = 0.0244/0.0189/0.1) [13]. The proteolysis conditions in that study differed from the conditions used in the present calculation, which may explain the difference in the estimates of this ratio.

The rate constants *k_d_* for the second demasking stage are apparently more complexly related to the structure of the polypeptide fragments. For β-LG proteolysis, the lowest *k_d_* = 0.32 was observed for the f(76–100/101) fragment, which is involved in the formation of the trypsin-resistant core [36]. For β-CN proteolysis, the lowest *k_d_* = 1 was determined for the central part and the hydrophobic C-terminus of β-CN f(106–209). A significant contribution of the hydrophobic C-terminal fragment to the demasking process was previously noted, explaining the formation of C-terminal peptides of β-CN with a lag [4].

The dependence of model parameters on enzyme and substrate concentrations is important for comparing and correctly interpreting experiments conducted under different kinetic conditions. We demonstrated how the contribution of secondary masking increases during proteolysis and how the concentration dependence changes with decreasing enzyme concentration at a constant substrate concentration [14]. However, experimental data are still insufficient to accurately determine the dependence of model parameters on proteolysis conditions, primarily on substrate concentration.

## 3. Discussion

The algorithm for calculating the release of peptides during proteolysis that we used is a new method that considers the need to demask peptide bonds before their hydrolysis [1,9]. The phenomenon of peptide bond demasking was first discovered based on the analysis of non-monotonic changes in the rate of peptide bond hydrolysis during enzymatic hydrolysis of milk protein substrates [4]. Then, the kinetics of demasking were studied by monitoring the fluorescence of tryptophan residues in the comparative analysis of β-LG and β-CN proteolysis by trypsin [13,14,17]. The importance of taking demasking processes into account in the study of proteolysis has also been demonstrated by other analytical and physicochemical methods [37,38].

It has previously been shown that accurate determination of demasking parameters may be even more important than precise determination of peptide bond hydrolysis rate constants [9]. In the previous works, the demasking rate constants were determined using a spectral method, which itself could introduce additional errors. To eliminate potential inaccuracies in determining demasking rate constants, in this study, demasking parameters were determined accurately using published experimental data [7] on the distribution of peptide fragments during proteolysis. Here, more accurate *k_d_* values were used for each ABC trimer under consideration, but the algorithm for calculating fragment concentrations was the same. The results of peptide release modeling showed better agreement with experiment than in the previous study [9].

All rate constants used in the model are first-order rate constants. The concentration of the active enzyme is included in these constants as a factor. Since it can change somewhat during proteolysis, the constancy of these constants can be approximate. However, the kinetics of demasking during proteolysis were studied previously, and it has been shown that this process obeys first-order kinetic law, and the demasking constants are indeed constant [13]. The use of first-order kinetics allows fragment concentrations to be expressed as analytical functions of time, significantly simplifying the calculations.

Using fluorescence spectroscopy, we have previously demonstrated that demasking of peptide bonds occurs differently during proteolysis of β-LG and β-CN by trypsin [1,17,39]. During proteolysis of β-LG, a rapid increase in the fluorescence wavelength (fluorescence red shift) is initially observed, followed by a slower red shift [1,13,14]. This corresponds to the opening of the protein globule in the first stage of demasking, followed by the demasking of the hydrolysis-resistant core of β-LG at the second stage. During proteolysis of β-CN, a rapid decrease in the fluorescence wavelength initially occurs, indicating an association of the polypeptide fragments of the hydrolysis of this substrate [17,39]. A continuous red shift in fluorescence is then observed, corresponding to the demasking and hydrolysis of peptide bonds that were masked at the beginning of proteolysis owing to association [17,39]. Despite the differences in the demasking processes for β-LG and β-CN, calculations using the same Equations (5)–(10) provide good agreement with experiment. Thus, the proposed calculation algorithm with more precise parameters correctly describes the release of peptide fragments even for structurally different protein substrates.

According to AFM, FTIR spectroscopy and static light scattering data, proteolysis of β-CN by trypsin is accompanied not only by degradation of the initial β-CN micelles but also by the parallel formation of new nanoparticles, which then degrade to form peptide proteolysis products [1,35,39]. When viewed at the macromolecular level, parallel degradation and association of intermediate polypeptide products are observed, with degradation gradually becoming predominant as the process progresses. New nanoparticles formed during proteolysis are denser than the initial micelles, and the content of secondary protein α- and β-structures in them does not decrease during proteolysis but is comparable to that in the initial β-CN micelles. In this regard, during proteolysis at low enzyme concentrations, the content of secondary protein structures changes non-monotonically. It first decreases due to hydrolysis of the original micelles, then increases due to the formation of these new nanoparticles, and finally slowly decreases as they are hydrolyzed [35,39]. The kinetics of degradation of β-CN micelles by trypsin were described by analytical functions in the case of modeling the proteolytic system using linear differential equations [35]. This process was also described by numerical modeling methods using nonlinear differential equations [1]. In this case, the original micelles, nanoparticles of various sizes and their aggregates, as well as change in the concentration of the active enzyme during proteolysis, were considered [1].

The macromolecular description of β-CN proteolysis by trypsin [35,39] and the model considered here represent sequential three-stage processes. A fairly rapid hydrolysis of micelles is the first stage in the macromolecular model and is initiated by the hydrolysis of the most specific peptide bonds [35]. Hydrolysis of the most specific peptide bonds also initiates the process of the first stage of demasking with rate constant $k_{d}^{f}$ in the model presented here. The ratio of the rate constant of micelle hydrolysis to the rate constant of peptide product formation in the macromolecular model was 33:1 [35]. A close value of 35 was obtained for the ratio of $k_{d}^{f}$ to *k^j^* for the peptide bonds with *j* = 107 and 113.

Relatively small β-CN and β-LG are used as substrates in model proteolytic reactions with various proteases, allowing the kinetic properties of this complex phenomenon to be studied [1]. For these substrates, the number of peptide fragments is not very large (several dozen), and they are easily identified and quantified using HPLC-MS [40,41,42,43,44,45]. Of particular importance in proteolysis studies are those in which the concentrations of peptide fragments are determined at several time points during proteolysis. Such data are indispensable for verifying proteolysis models and determining the numerical values of kinetic parameters. In particular, such studies have been carried out for the proteolysis of β-CN [7] and β-LG by trypsin [7,8,10]. Quantification of the individual peptides made it possible to determine the changes in the concentrations of peptide bonds during proteolysis and then to determine the selectivity parameter for different peptide bonds [7]. In this study, the summation of the concentrations of peptides ABC, AB, and A (ABC, BC and C) was used to determine the demasking rate constants. In our opinion, this method is as important as the determination of selectivity parameters, since accurate determination of demasking parameters is important for proteolysis modeling [1,9].

Although the spectroscopic methods were not used in this work, these methods have great potential for the monitoring of protein opening during proteolysis and hence for the monitoring of demasking. The possibility of the monitoring of protein structure changes in the course of proteolysis was demonstrated using fluorescence [13], infrared (FTIR) [35], ultrasound [46,47,48] and other physicochemical methods. Undoubtedly, further development of these analytical methods is of great importance for successful modeling of proteolysis.

In practically oriented proteolysis models, the peptide bonds and their hydrolysis rate constants are assumed to be identical, and the main model variables are the degree of the peptide bond hydrolysis and the total hydrolysis rate [49,50,51,52,53,54]. Such models can be called models of total proteolysis, since they do not take into account the differences in hydrolysis of different peptide bonds. The model we are developing is more accurate because it considers differences in the hydrolysis of various peptide bonds in proteins, as well as the limited accessibility of the enzyme to some temporarily masked regions of the polypeptide chains [1,9]. Complete proteolysis models can be used to optimize the production of protein hydrolysate with the desired degree of hydrolysis, while our approach should be used to optimize the yield of target biopeptides.

The quality of published experimental data on peptide fragments [7] is determined both by the coverage of the protein amino acid sequence by the identified peptides and by the accuracy of determining the molar concentrations of peptides using UV detection of chromatographic peaks. The molar concentrations of peptides were determined using the molar extinction coefficients predicted from their amino acid sequence [55]. This method significantly simplifies the quantification of molar concentrations but also introduces additional errors [55]. The proteolysis parameters *d_r_* and *n* were introduced by Equations (1) and (2) such that they did not depend on the absolute molar concentrations of the peptide components but depended on the relative ones. This reduced the impact of possible errors in determining the absolute molar concentrations of peptides on the proteolysis modeling results.

For β-LG hydrolysates, the peptide sequence coverage was 90%, whereas for β-CN hydrolysates, the peptide sequence coverage averaged 83% among the hydrolysates analyzed [7]. This parameter was highest at the beginning of proteolysis and decreased with increasing degree of hydrolysis. This is explained by the increase in the number of peptide fragments with increasing degree of hydrolysis and, consequently, an increase in the probability of overlapping chromatographic peaks [30]. Peptide sequence coverage for β-CN hydrolysates was poorer than for β-LG hydrolysates obtained with both trypsin [7] and chymotrypsin [41]. In addition to the amphiphilicity of β-CN and the tendency of peptide fragments to aggregate, the lower peptide sequence coverage for β-CN was also explained by the presence of phosphoseryl N-terminal peptide fragments of β-CN, which are typically not well ionized [7]. The better peptide sequence coverage for β-LG hydrolysates apparently resulted in a better correlation between the predicted and experimental model parameters with *r*^2^ = 0.9998 (Figure 4a). Overall, despite some limitations of the analytical method used in [7], our study demonstrates that published experimental data are sufficient to validate the general model of proteolysis (Figure 1). Other research groups can use the presented algorithm and determine the demasking and hydrolysis rate constants for other protein substrates and proteases.

Although our study demonstrated a relationship between some structural features of the substrates used and demasking parameters, reliable correlation equations that could be used to predict proteolysis parameters for any protein substrate are not yet available. To determine the parameters of the correlation equations, it is necessary to have much more data than is presented in Figure 4b. Since in silico proteolysis of various protein substrates is impossible without knowledge of the model parameters, a hybrid approach can be proposed in which some parameters are determined experimentally. For example, in the calculations presented here, the demasking parameter *k_d_* was determined by analyzing experimental data using Equation (12), and the identification of rapidly hydrolyzed bonds was also based on the experimental data (Table 1). The success of the hybrid approach is possible by minimizing the number of experimentally determined parameters. For such a complex phenomenon as proteolysis, the number of experimental parameters used in our model is apparently not so large.

We hope that the use of this approach will help bridge the gap between the experimental study of specific proteolytic processes and the development of a general proteolysis model.

## 4. Materials and Methods

### 4.1. Quantitative Modeling of Proteolysis

The following system of ordinary differential equations for the concentrations of the fragments [-*ABC*-], [-*ABC*], [*ABC*], [*AB*], [*BC*], [*A*], [*B*], and [*C*] corresponds to the general scheme of proteolysis (Figure 1):

(3) $$\begin{array}{l} \frac{d[-ABC-]}{dt}=-k_{d}^{f}[-ABC-]\\ \frac{d[-ABC]}{dt}=k_{d}^{f}[-ABC-]-k_{d}[-ABC]\\ \frac{d[ABC]}{dt}=k_{d}[-ABC]-(k^{i}+k^{j})[ABC]\\ \frac{d[AB]}{dt}=k^{j}[ABC]-k^{i}[AB]\\ \frac{d[BC]}{dt}=k^{i}[ABC]-k^{j}[BC]\\ \frac{d[A]}{dt}=k^{i}[ABC]+k^{i}[AB]\\ \frac{d[B]}{dt}=k^{i}[AB]+k^{j}[BC]\\ \frac{d[C]}{dt}=k^{j}[ABC]+k^{j}[BC] \end{array}$$

For each amino acid sequence A, B or C, there is a material balance equation; for example, for A units, it looks like this:

(4) [−ABC−]+[−AB]+[ABC]+[AB]+[A]=1

The solutions of the differential equations corresponding to the proteolysis scheme (Figure 1) were found as analytical functions of the hydrolysis time *t*, min. The concentrations *C*(*t*) of the peptide fragments ABC, AB, BC, A, B, and C are equal to:

(5) $$[ABC]=\frac{k_{d}^{f}k_{d}}{\left(k_{d}-k_{d}^{f}\right)\left(k^{i}+k^{j}-k_{d}^{f}\right)}e^{-k_{d}^{f}t}+\frac{k_{d}^{f}k_{d}}{\left(k^{i}+k^{j}-k_{d}^{f}\right)\left(k^{i}+k^{j}-k_{d}\right)}e^{-(k^{i}+k^{j})t}-\frac{k_{d}^{f}k_{d}}{\left(k_{d}-k_{d}^{f}\right)\left(k^{i}+k^{j}-k_{d}\right)}e^{-k_{d}t}$$

(6) $$\begin{array}{l} {}[AB]=\frac{k_{d}^{f}k_{d}k^{j}}{\left(k_{d}-k_{d}^{f}\right)\left(k^{i}+k^{j}-k_{d}^{f}\right)\left(k^{i}-k_{d}^{f}\right)}e^{-k_{d}^{f}t}-\frac{k_{d}^{f}k_{d}}{\left(k^{i}+k^{j}-k_{d}^{f}\right)\left(k^{i}+k^{j}-k_{d}\right)}e^{-\left(k^{i}+k^{j}\right)t}-\frac{k_{d}^{f}k_{d}k^{j}}{\left(k_{d}-k_{d}^{f}\right)\left(k^{i}+k^{j}-k_{d}\right)\left(k^{i}-k_{d}\right)}e^{-k_{d}t}+\\ \frac{k_{d}^{f}k_{d}\left[\left(k^{i}+k^{j}\right)\left(k^{i}+k^{j}-k_{d}^{f}-k_{d}\right)+k_{d}^{f}k_{d}\right]}{\left(k^{i}-k_{d}^{f}\right)\left(k^{i}-k_{d}\right)\left(k^{i}+k^{j}-k_{d}^{f}\right)\left(k^{i}+k^{j}-k_{d}\right)}e^{-k^{i}t} \end{array}$$

(7) $$\begin{array}{l} {}[BC]=\frac{k_{d}^{f}k_{d}k^{i}}{\left(k_{d}-k_{d}^{f}\right)\left(k^{i}+k^{j}-k_{d}^{f}\right)\left(k^{j}-k_{d}^{f}\right)}e^{-k_{d}^{f}t}-\frac{k_{d}^{f}k_{d}}{\left(k^{i}+k^{j}-k_{d}^{f}\right)\left(k^{i}+k^{j}-k_{d}\right)}e^{-\left(k^{i}+k^{j}\right)t}-\frac{k_{d}^{f}k_{d}k^{i}}{\left(k_{d}-k_{d}^{f}\right)\left(k^{i}+k^{j}-k_{d}\right)\left(k^{j}-k_{d}\right)}e^{-k_{d}t}\\ +\frac{k_{d}^{f}k_{d}\left[\left(k^{i}+k^{j}\right)\left(k^{i}+k^{j}-k_{d}^{f}-k_{d}\right)+k_{d}^{f}k_{d}\right]}{\left(k^{j}-k_{d}^{f}\right)\left(k^{j}-k_{d}\right)\left(k^{i}+k^{j}-k_{d}^{f}\right)\left(k^{i}+k^{j}-k_{d}\right)}e^{-k^{j}t} \end{array}$$

(8) $$[A]=1-\frac{k^{i}k_{d}}{\left(k_{d}-k_{d}^{f}\right)\left(k^{i}-k_{d}^{f}\right)}e^{-k_{d}^{f}t}+\frac{k_{d}^{f}k^{i}}{\left(k_{d}-k_{d}^{f}\right)\left(k^{i}-k_{d}\right)}e^{-k_{d}t}-\frac{k_{d}^{f}k_{d}\left[\left(k^{i}+k^{j}\right)\left(k^{i}+k^{j}-k_{d}^{f}-k_{d}\right)+k_{d}^{f}k_{d}\right]}{\left(k^{i}-k_{d}^{f}\right)\left(k^{i}-k_{d}\right)\left(k^{i}+k^{j}-k_{d}^{f}\right)\left(k^{i}+k^{j}-k_{d}\right)}e^{-k^{i}t}$$

(9) $$\begin{array}{l} {}[B]=1-\frac{k_{d}k^{i}k^{j}(k^{i}+k^{j}-2k_{d}^{f})}{\left(k_{d}-k_{d}^{f}\right)\left(k^{i}+k^{j}-k_{d}^{f}\right)\left(k^{j}-k_{d}^{f}\right)\left(k^{i}-k_{d}^{f}\right)}e^{-k_{d}^{f}t}+\frac{k_{d}^{f}k_{d}}{\left(k^{i}+k^{j}-k_{d}^{f}\right)\left(k^{i}+k^{j}-k_{d}\right)}e^{-\left(k^{i}+k^{j}\right)t}\\ +\frac{k_{d}^{f}k^{i}k^{j}(k^{i}+k^{j}-2k_{d})}{\left(k_{d}-k_{d}^{f}\right)\left(k^{i}+k^{j}-k_{d}\right)\left(k^{j}-k_{d}\right)\left(k^{i}-k_{d}\right)}e^{-k_{d}t}-\frac{k_{d}^{f}k_{d}\left[\left(k^{i}+k^{j}\right)\left(k^{i}+k^{j}-k_{d}^{f}-k_{d}\right)+k_{d}^{f}k_{d}\right]}{\left(k^{i}-k_{d}^{f}\right)\left(k^{i}-k_{d}\right)\left(k^{i}+k^{j}-k_{d}^{f}\right)\left(k^{i}+k^{j}-k_{d}\right)}e^{-k^{i}t}\\ -\frac{k_{d}^{f}k_{d}\left[\left(k^{i}+k^{j}\right)\left(k^{i}+k^{j}-k_{d}^{f}-k_{d}\right)+k_{d}^{f}k_{d}\right]}{\left(k^{j}-k_{d}^{f}\right)\left(k^{j}-k_{d}\right)\left(k^{i}+k^{j}-k_{d}^{f}\right)\left(k^{i}+k^{j}-k_{d}\right)}e^{-k^{j}t} \end{array}$$

(10) $$[C]=1-\frac{k^{j}k_{d}}{\left(k_{d}-k_{d}^{f}\right)\left(k^{j}-k_{d}^{f}\right)}e^{-k_{d}^{f}t}+\frac{k_{d}^{f}k^{j}}{\left(k_{d}-k_{d}^{f}\right)\left(k^{j}-k_{d}\right)}e^{-k_{d}t}-\frac{k_{d}^{f}k_{d}\left[\left(k^{i}+k^{j}\right)\left(k^{i}+k^{j}-k_{d}^{f}-k_{d}\right)+k_{d}^{f}k_{d}\right]}{\left(k^{j}-k_{d}^{f}\right)\left(k^{j}-k_{d}\right)\left(k^{i}+k^{j}-k_{d}^{f}\right)\left(k^{i}+k^{j}-k_{d}\right)}e^{-k^{j}t}$$

In the calculations, $k_{d}^{f}$ was 0.46 min^−1^ [9] for β-LG and 3.5 min^−1^ for β-CN; the other rate constants (*k_d_* and *k^i^*) were taken from Table 2. When $k_{d}^{f}$ >> *k_d_*, the two-stage demasking turns into one-stage demasking and Equations (5)–(10) are transformed into Equations (1)–(6), given in [9].

### 4.2. Estimation of the Rate Constants

The parameter $k_{d}^{f}$ was the largest of the hydrolysis rate constants for the most rapidly hydrolyzed peptide bonds. These constants were determined using a simple equation for the dependence of the concentration *N^j^*(*t*) of peptide bond *j* on hydrolysis time *t*:

(11) $$N^{j}(t)=N_{0}^{j}\left(1-e^{-k^{j}t}\right)$$

By summing the concentrations of peptide fragments ABC, AB and A from Equations (5), (6) and (8), we obtained the following equation for the sum [*ABC*] + [*AB*] + [*A*]:

(12) $$[ABC]+[AB]+[A]=A_{0}\left[1-\frac{k_{d}e^{-k_{d}^{f}t}}{\left(k_{d}-k_{d}^{f}\right)}+\frac{k_{d}^{f}e^{-k_{d}t}}{\left(k_{d}-k_{d}^{f}\right)}\right]$$

A similar equation was obtained for the sum [*ABC*] + [*BC*] + [*C*]. The dependence of the sums of peptide fragments [*ABC*] + [*AB*] + [*A*] and [*ABC*] + [*BC*] + [*C*] on the hydrolysis time were calculated from the published data [7] and used to estimate the parameter *k_d_* with Equation (12).

The parameters $k_{d}^{f}$ and *k_d_* were then used to calculate the rate constants of hydrolysis *k^j^* for different peptide bonds *j* using the following equation expressing the dependence of the peptide bond concentration for the j-th bond on the hydrolysis time [1,14]:

(13) $$N^{j}(t)=N_{0}^{j}\left[1-\frac{k_{d}k^{j}e^{-k_{d}^{f}t}}{\left(k_{d}-k_{d}^{f})(k^{j}-k_{d}^{f}\right)}-\frac{k_{d}^{f}k^{j}e^{-k_{d}t}}{\left(k_{d}-k_{d}^{f})(k_{d}-k^{j}\right)}-\frac{k_{d}^{f}k_{d}e^{-k^{j}t}}{\left(k^{j}-k_{d}^{f})(k^{j}-k_{d}\right)}\right]$$

The values of *k_d_*, and *k^j^* were calculated by the method of nonlinear least squares fitting with program Origin (v8.5.1, OriginLab Corp., Northampton, MA, USA). Origin’s Nonlinear Curve Fit tool was engaged, and the user functions were specified as Equation (12) to determine *k_d_* and Equation (13) to determine *k^j^*. The assignment of the analyzed trimers to one-stage or two-stage demasking follows from the result of fitting using Equation (12). When the fitting program yields a *k_d_* value significantly greater than $k_{d}^{f}$, this indicates that the second step is kinetically insignificant, and demasking occurs via one-stage demasking.

The experimental concentrations of peptide fragments taken from [7] were divided by a factor of 50 to obtain the relative concentrations of the peptide fragments that were used in the calculation. The values of the model parameters *d_r_* and *n* were independent of this factor.

## Figures and Tables

**Table 1:** Kinetic characterization of tryptic proteolysis of β-CN.

Bond Index *i*	Cleavage Site ^1^ $P_{2}P_{1}\frac{↓}{\;}P_{-1}P_{-2}$	Selectivity ^2^ (%)	Initial Hydrolysis Rate ^2^	Most Rapidly Hydrolyzed Bonds	Most Slowly Hydrolyzed Bonds	Peptide Fragments in Trimer
1	R-EI	0	0		+	1–28/29, 30–97, 98–99
25	TR-IN	0.7	0		+	
28/29	NK-KI/KK-IE	8.2/0.9	0.1			
32	EK-FQ	0.7	0		+	
48	DK-IH	0.02	0		+	
97	EK-TK	0.6	0.1			
99	VK-EA	15.3	0.8	+		
105	PK-HK	23.4	0.8	+		106–107, 108–113, 114–169
107	HK-EM	2.7	0.3			
113	PK-YP	1.0	0.05			
169	SK-VL	32.4	1	+		
176	QK-AV	11.4	0.6			170–176, 177–183, 184–209
183	QR-DM	2.8	0.2			
202	VR-GP	0.2	0		+	

^1^ Peptide bonds 28 and 29 in the amino acid sequence -Lys_28_-Lys_29_- were considered as one hydrolysis site with single Lys bond in P_1_ position Lys-X and denoted as cleavage site 28/29. ^2^ The values of enzyme selectivity and initial hydrolysis rate were from [7].

**Table 2:** Demasking and hydrolysis rate constants for tryptic proteolysis of β-CN and β-LG.

Substrate	Bond Index *i*	$k_{d}^{f}$ (min^−1^)	*k_d_* (min^−1^) ^2^	*k^i^* (min^−1^) ^3^	$N_{0}^{i}$ ^3^
	28/29	3.5	5	0.6 ± 0.2	70 ± 3
	97	3.5	5	0.3 ± 0.1	32 ± 2
	99	Most rapidly hydrolyzed bond			
	105	Most rapidly hydrolyzed bond			
β-CN	107	3.5	1	0.10 ± 0.01	19 ± 1
	113	3.5	1	0.10 ± 0.01	75 ± 3
	169	Most rapidly hydrolyzed bond			
	176	3.5	1	10 ± 1	78 ± 5
	183	3.5	1	0.3 ± 0.1	80 ± 8
	8	Most rapidly hydrolyzed bond			
	14	0.46	>>1	0.9 ± 0.2	93 ± 7
	40	0.46	>>1	1.6 ± 0.4	102 ± 5
	69/70	Most rapidly hydrolyzed bond			
β-LG ^1^	75	Most rapidly hydrolyzed bond			
	83	0.46	0.32	0.6 ± 0.1	115 ± 8
	91	0.46	0.32	1.1 ± 0.3	96 ± 3
	100/101	Most rapidly hydrolyzed bond			
	124	0.46	1.1	0.55 ± 0.14	80 ± 6
	135	0.46	1.1	0.05 ± 0.01	19 ± 1
	138	Most rapidly hydrolyzed bond			
	141	Most rapidly hydrolyzed bond			
	148	Most rapidly hydrolyzed bond			

^1^ The β-LG cleavage sites were taken to be the same as in [9]. ^2^ The rate constants *k_d_* were determined using Equation (12). ^3^ The rate constants *k^i^* and amplitudes *N*_0_ were determined using Equation (13).

**Table 3:** Modeling of peptide release during tryptic proteolysis of β-CN and β-LG in comparison with experiment.

Substrate	Peptide	Type of Fragment	Calculated *d_r_* (%) ^1^	Experimental *d_r_* (%) ^1^	Calculated *n* ^2^	Experimental *n* ^2^
	f(1–99), ABC	Intermediate	1.41	1.00		
	f(1–97), AB	Intermediate	2.04	1.77		
	f(30–99), BC	Intermediate	2.23	2.28		
	f(106–169), ABC	Intermediate	2,18	2.14		
	f(106–113), AB	Intermediate	2.98	3.61		
	f(108–169), BC	Intermediate	2.98	1.96		
	f(170–209), ABC	Intermediate	1.32	1.33		
	f(170–183), AB	Intermediate	1.40	1.48		
	f(177–209), BC	Intermediate	2.14	1.61		
	f(1–28/29), A	Final			1.01	1.10
β-CN	f(30–97), B	Final			1.75	2.10
	f(98–99), C	Final			1.42	- ^3^
	f(106–107), A	Final			3.11	- ^3^
	f(108–113), B	Final			4.78	5.26
	f(114–169), C	Final			3.11	2.98
	f(170–176), A	Final			0.74	0.55
	f(177–183), B	Final			1.79	1.31
	f(184–209), C	Final			1.78	2.00
	f(9–69/70), ABC	Intermediate	2.45	1.50 ^3^		
	f(9–40), AB	Intermediate	2.90	3.60		
	f(15–69/70), BC	Intermediate	2.65	- ^3^		
	f(76–100/101), ABC	Intermediate	3.62	3.40		
	f(76–91), AB	Intermediate	4.29	4.40		
	f(84–100/101), BC	Intermediate	4.06	4.70		
β-LG	f(101/102–138),ABC	Intermediate	3.41	3.40		
	f(101/102–135), AB	Intermediate	4.20	- ^3^		
	f(125–138), BC	Intermediate	4.85	6.10		
	f(9–14), A	Final			0.70	0.68
	f(15–40), B	Final			0.78	0.86
	f(41–69/70), C	Final			0.56	0.59
	f(76–83), A	Final			1.83	1.46
	f(84–91), B	Final			2.03	2.37
	f(92–100/101), C	Final			1.52	1.40
	f(101/102–124), A	Final			1.18	1.13
	f(125–135), B	Final			5.70	5.76
	f(136–138), C	Final			5.29	5.10

^1^ The parameter *d_r_* was determined using Equation (1). ^2^ The exponent of the power function *n* was determined using Equation (2). ^3^ These peptide fragments were not found in the hydrolysate [7].

## Data Availability

The data presented in this study are available on request from the corresponding author.
